# Supplementary material for: Artificial hibernation/life-protective state induced by thiazoline-related innate fear odors
Source: Commun Biol. 2021 Jan 22;4:101. doi: 10.1038/s42003-020-01629-2 (PMC7822961; doi:10.1038/s42003-020-01629-2)
Supplement: Supplementary file 2 — Supplementary Information [file 42003_2020_1629_MOESM2_ESM.pdf]

Supplementary Information

Artificial hibernation/life-protective state induced by thiazoline-related innate fear odors

Tomohiko Matsuo, Tomoko Isosaka, Lijun Tang, Tomoyoshi Soga, Reiko Kobayakawa, Ko Kobayakawa

Supplementary Figure 1. Suppressed locomotor activities during long exposure to 2MT

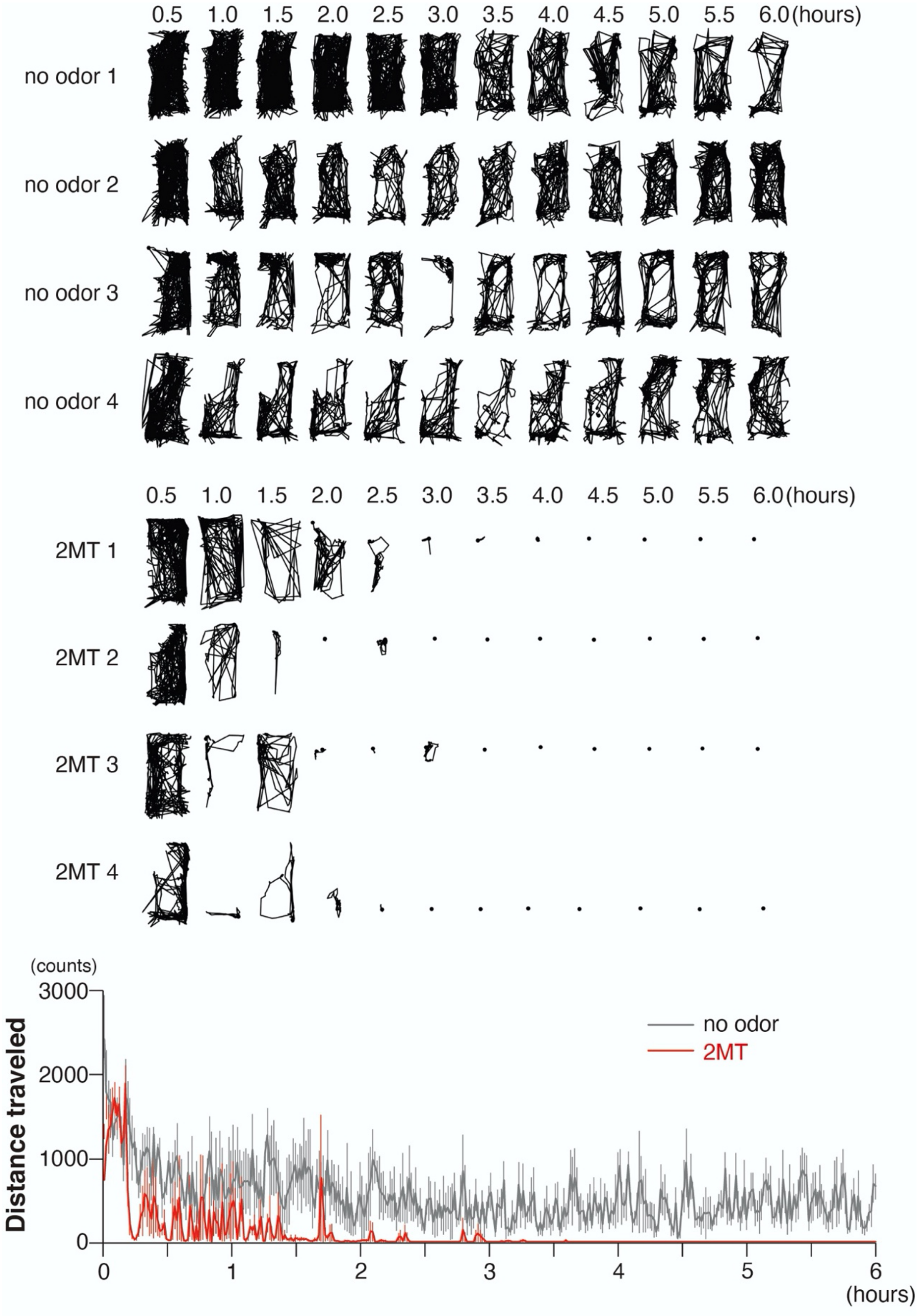

Filter papers scented with or without 2MT were introduced into the cage, and locomotor activities were analyzed during 6 h of exposure to 2MT (filter papers were presented at 10 min). Track plots of the movement (upper panels) and the mean distance traveled (lower panels) are shown.

Supplementary Figure 2. Effects of restraint, learned fear stimuli and corticosterone administration on anti-hypoxia

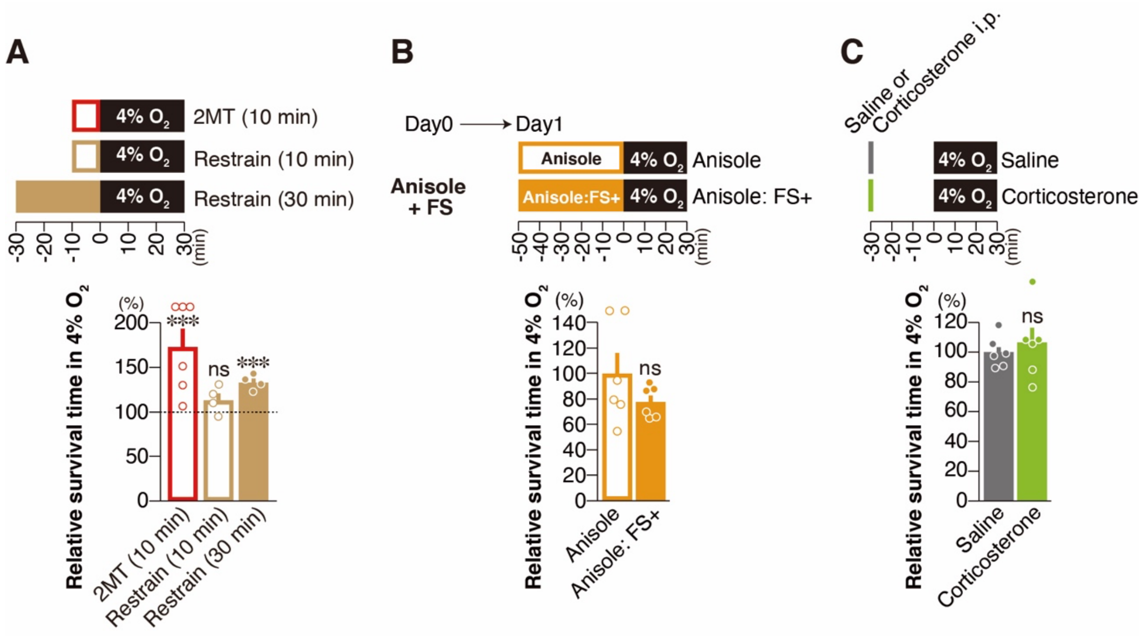

(**A**) Mean survival times in 4% oxygen with prior stimulation with 10 min 2MT presentation (red; n = 6 each) and with 10 min or 30 min restraint (brown; n = 4 each). Survival times without odor presentation or restraint were set at 100%.

(**B**) Mean survival time in 4% oxygen with prior stimulation with 50 min of anisole previously paired with electric shocks (n = 6). Mean survival time with prior stimulation with anisole for control group (without conditioning; n = 6) was set at 100%.

(**C**) Mean survival times in 4% oxygen with and without 30 min prior IP administration of 2mg/kg corticosterone, which corresponds to physiological concentration detected in stressed mice [1-3] (n = 6 each). Survival time without corticosterone administration was set at 100%.

The experimental timelines are shown on the top. Data are means ± SEM. Unpaired one-tailed student's t-test or one-way ANOA followed by Dunnett's multiple comparison was performed between control and each condition. ***$p<0.001$; ns, $p>0.05$.

Supplementary Figure 3. Effective concentration of tFOs to confer anti-hypoxia

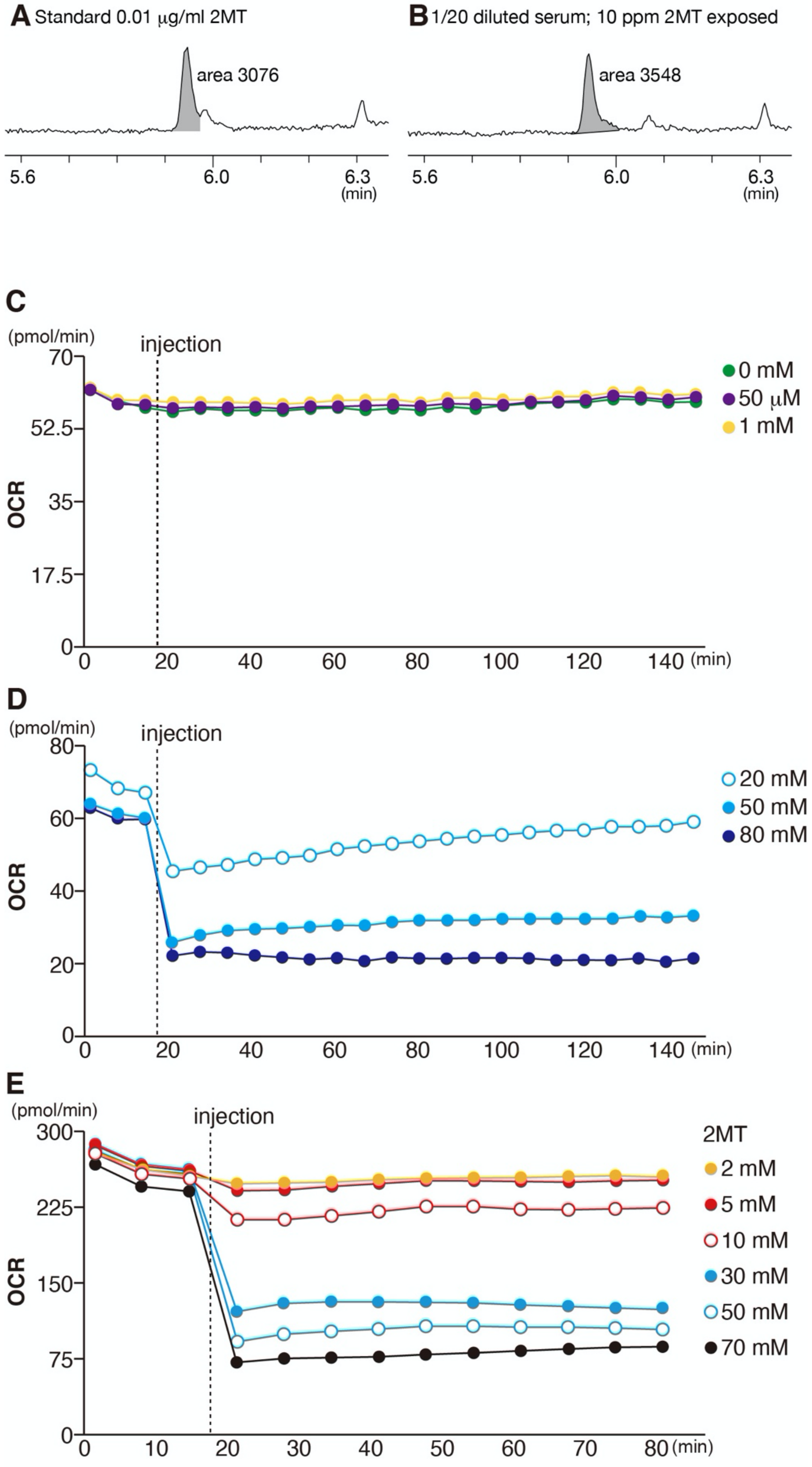

(**A** and **B**) Extracted ion chromatograms (m/z 60) are shown for standard 0.01 μg/ml 2MT solution (**A**) and serum sample (**B**). The peak areas (gray) are also shown.

(**C**-**E**) Oxygen consumption rate (OCR) in A549 (**C**, **D**) and HepG2 (**E**) cells in response to indicated concentrations of 2MT. 2MT concentrations up to 2mM did not affect OCR in HepG2 cells.

Supplementary Figure 4. Metabolic changes in the liver induced by innate fear stimuli

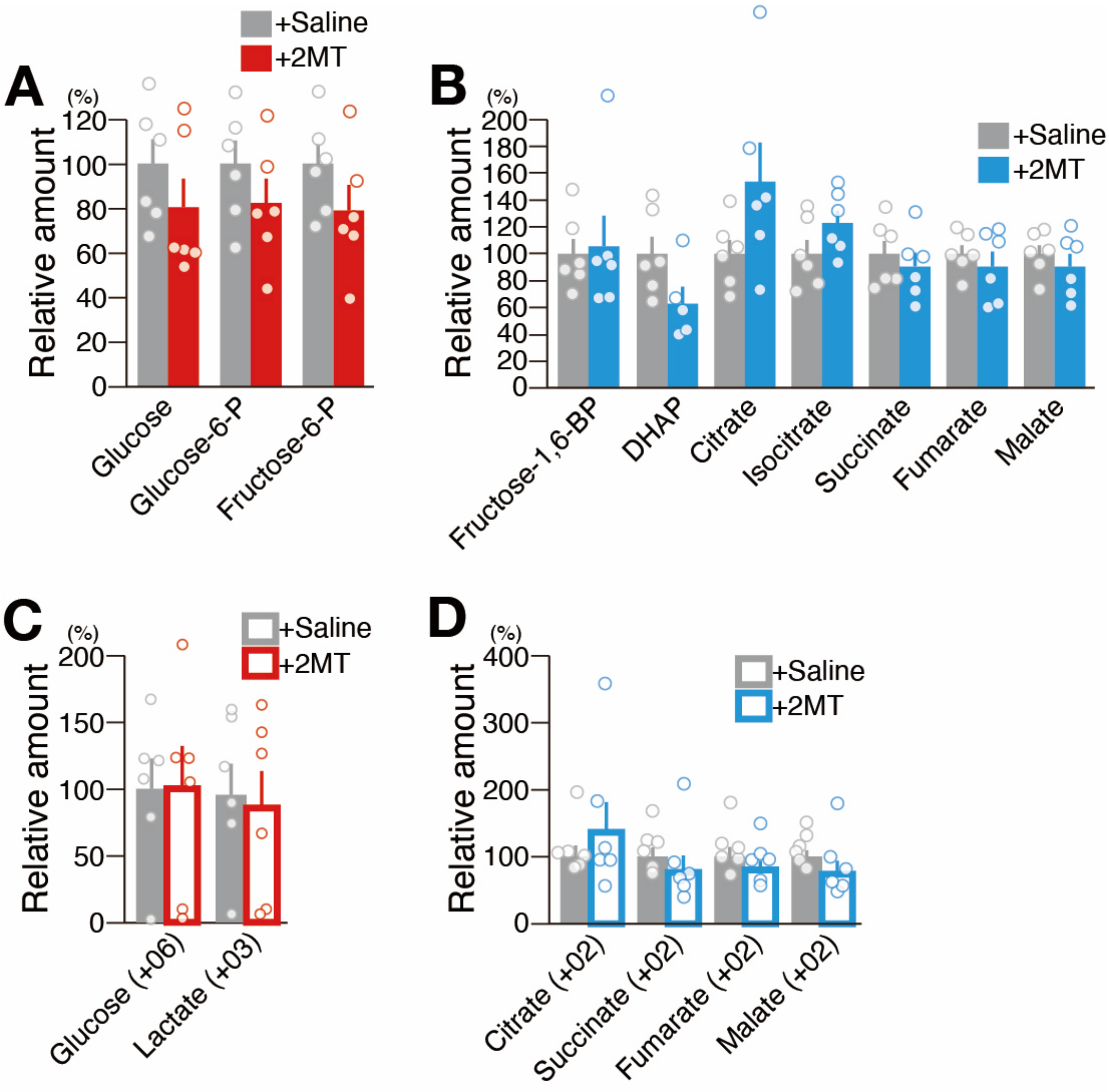

Mean percentages of ^{13}C-unlabelled (**A** and **B**) and ^{13}C-labelled (**C** and **D**) metabolites in response to saline (gray) and 2MT (red or blue) (n = 6 each). Metabolite levels in response to saline injection were set at 100%. 2MT stimulation did not induce significant effects on either glycolysis or TCA cycle activity in the liver. Data are means ± SEM.

Supplementary Figure 5. Brain activation and fear-related behavioral/physiological responses induced by odor presentation and IP injection of tFOs

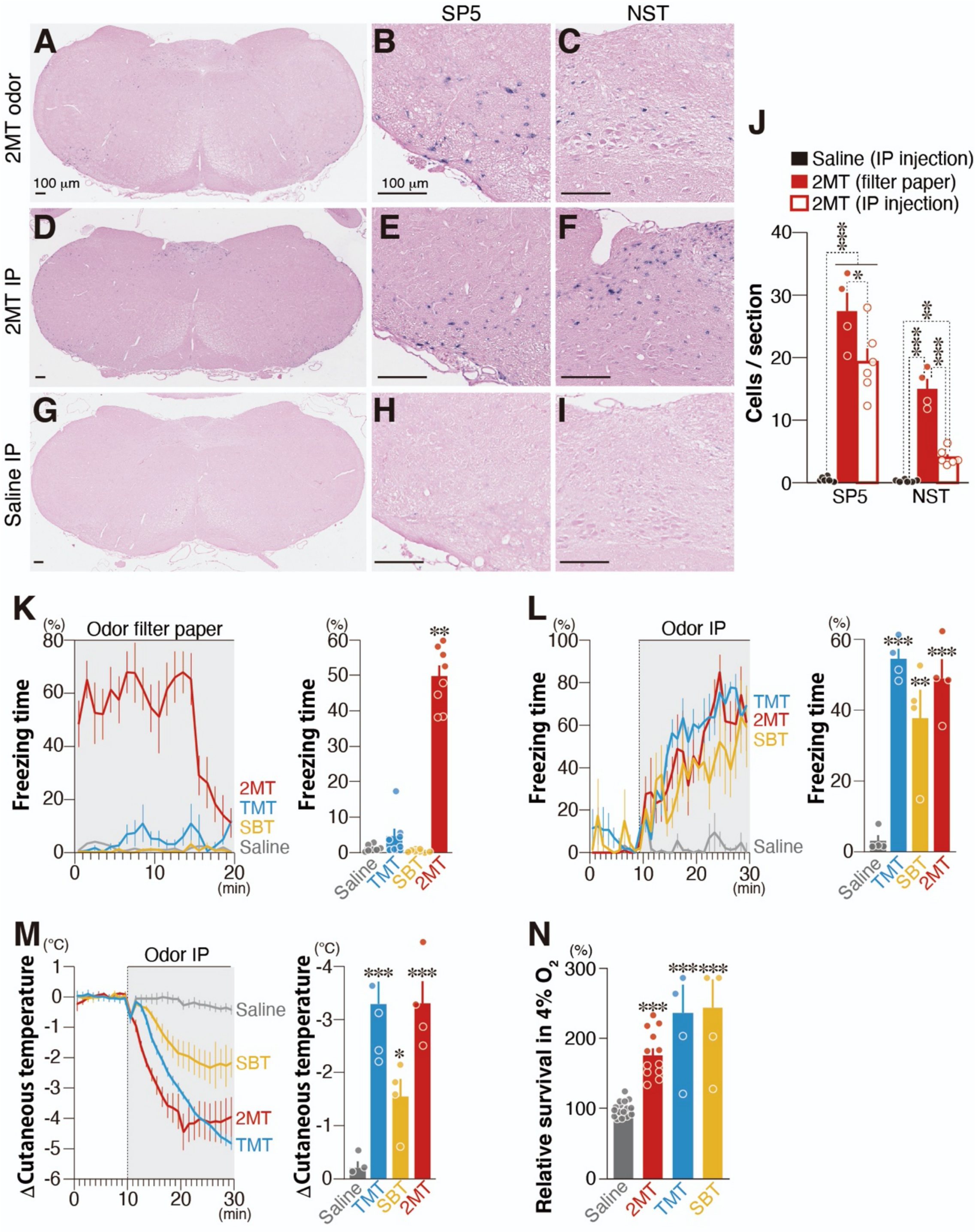

(**A**–**J**) Representative images of *in situ* hybridization for *c-fos* mRNA in the spinal trigeminal nucleus (SP5) and nucleus of the solitary tract (NST) following odor exposure to 2MT (**A**–**C**), and

intraperitoneal (IP) injection of 2MT (**D**–**F**) and saline (**G**–**I**), along with enlarged images of the SP5 (**B**, **E**, and **H**) and NST (**C**, **F**, and **I**). Quantified *c-fos*$^{+}$ cells are also shown (**J**; n = 6 for saline IP and 2MT IP; n = 4 for 2MT filter paper).

(**K** and **L**) Temporal analyses (left panels) and mean levels of freezing behaviors in response to odor presentation (**K**) and intraperitoneal (IP) injection (**L**) of 2MT, 2,4,5-trimethyl-3-thiazoline (TMT), 2-*sec*-butyl-2-thiazoline (SBT) and saline (**K**, n = 6 for saline and n = 8 for test odorants; **L**, n = 4 each).

(**M**) Temporal analyses of cutaneous temperature in response to IP injection of the indicated odorants (n = 4 each).

(**N**) Mean survival times in 4% oxygen following IP injection of the indicated odorants (n = 12 for 2MT, n = 4 for SBT, TMT). Mean survival time following saline injection was set at 100%.

Data are means ± SEM. One-way ANOVA followed by Tukey's multiple comparison test, Kruskal-Wallis followed by Dunn's multiple comparison, or one-way ANOVA followed by Dunnett's multiple comparison test was performed. *p<0.05, **p<0.01, ***p<0.001; ns, p>0.05.

Vaporized odor presentations of TMT, a component of fox feces, and SBT, an endogenous chemical that acts as an alarm pheromone in mice, induced only weak or no freezing behavior. However, intraperitoneally injected, these odorants induced robust freezing behavior and hypothermia, and extended survival time under hypoxic condition.

Supplementary Figure 6. *C-fos* mRNA expression analysis in the PBN

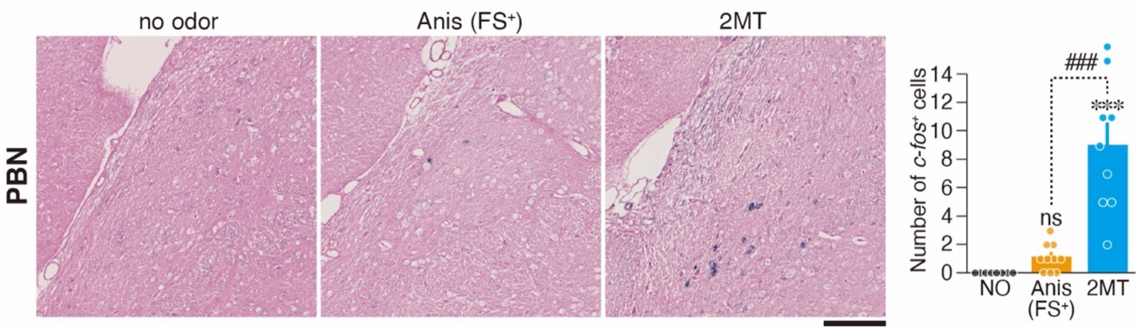

Representative images of *in situ* hybridization of *c-fos* mRNA in response to no odor, Anis(FS+), and 2MT in the PBN [n = 8 for no odor, n = 10 for Anis(FS+), and n = 9 for 2MT]. Quantification of *c-fos*$^{+}$ cells are also shown.

Data are means ± SEM. One-way ANOVA followed by Tukey's multiple comparison was performed. ***p<0.001; #p<0.001; ns, p>0.05.

Supplementary Figure 7. Chemogenetic activation of NST-PBN pathway

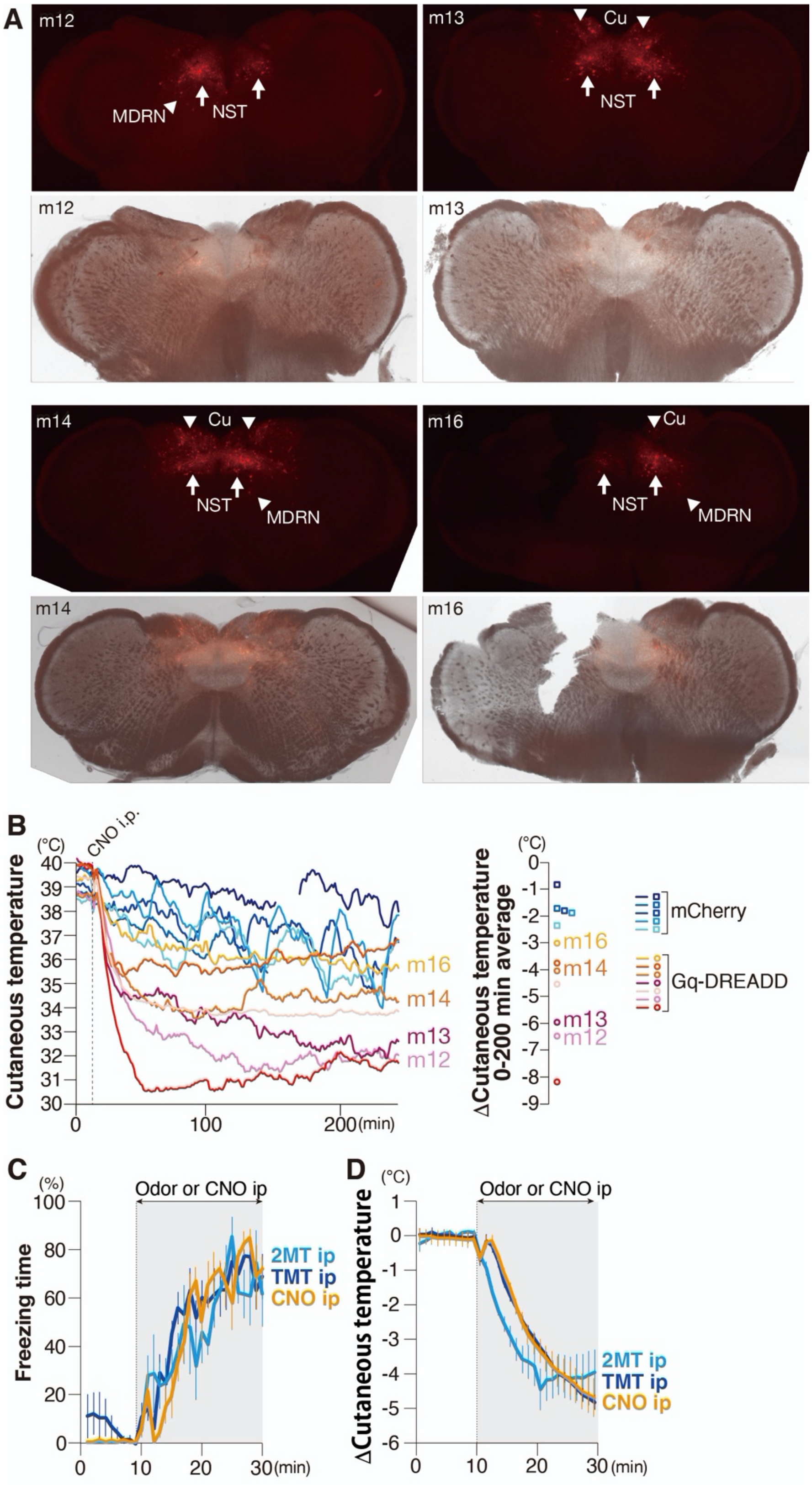

(**A**) Representative fluorescent (top) and bright (bottom) images of the NST for four AAV-FLEX-hM3Dq-mCherry infected animals (m12, m13, m14 and m16).
(**B**) Temporal (left) and mean (right) cutaneous temperature in response to CNO administration for AAV-FLEX-mCherry (mCherry) and AAV-FLEX-hM3Dq-mCherry infected animals.
(**C** and **D**) Temporal analysis of freezing behavior (**C**) and cutaneous temperature (**D**) in response to CNO administration for AAV-FLEX-hM3Dq-mCherry infected animals were compared to those for C57/BL6 mice IP injected with 2MT or TMT.

Supplementary Figure 8. Chemogenetic and pharmacological inactivation of NST-PBN pathway

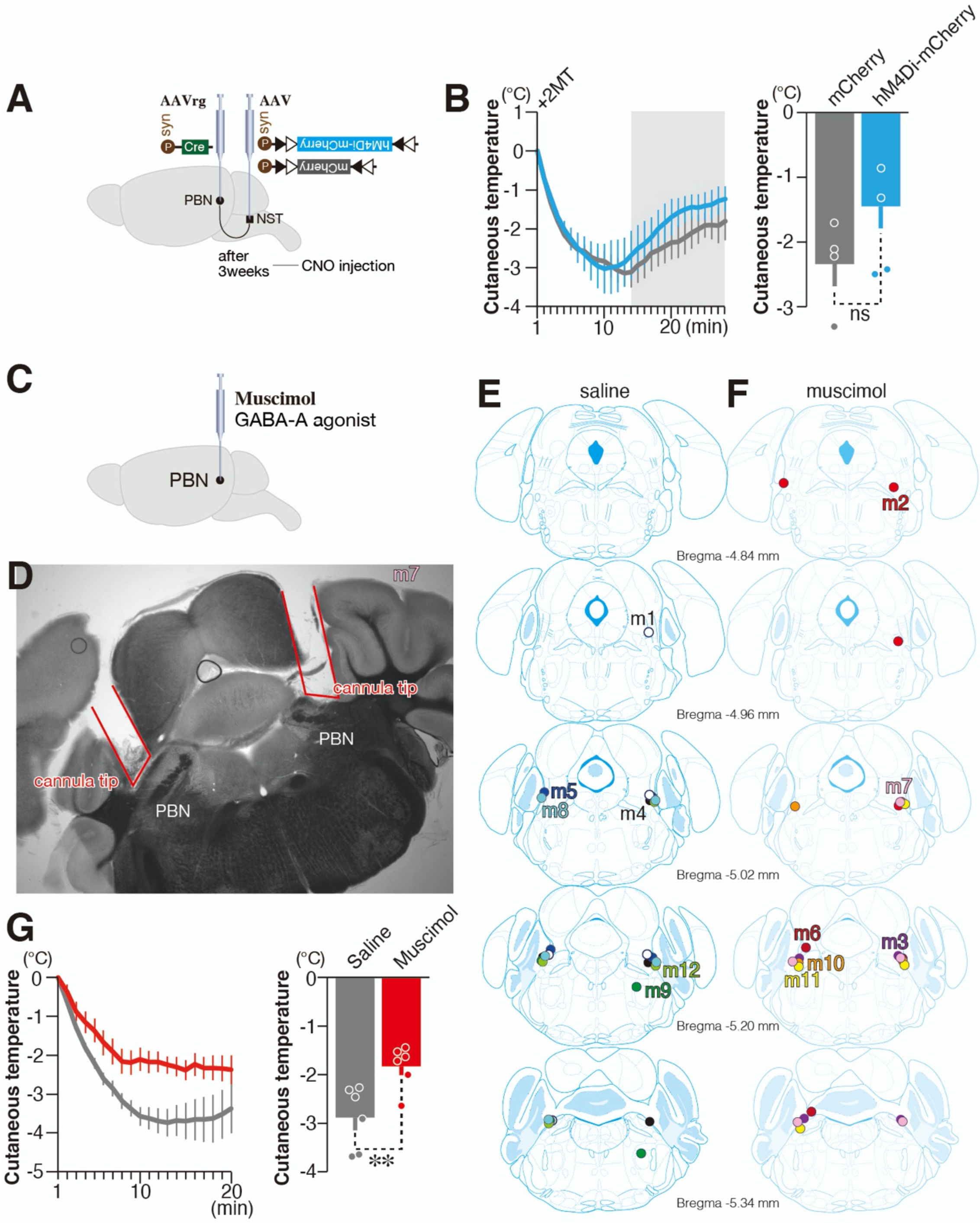

(**A**) Experimental design for chemogenetic inhibition of the NST-PBN pathway is shown. 2MT odor was presented 40 min after IP injection of CNO.

(**B**) Temporal analyses of cutaneous temperature changes during 30 min of 2MT presentation (left) and the mean cutaneous temperature changes during gray shaded duration (15-30 min after 2MT

presentation) are indicated (right). The cutaneous temperature change was calculated as the change from the onset of 2MT presentation (n = 4 each).

(**C**) Experimental design for pharmacological inhibition of the NST-PBN pathway is shown. 2MT was presented 15 min after muscimol infusion into the PBN.

(**D**) Representative image of the cannula placement in the PBN.

(**E**, **F**) The locations of cannula tips were verified by examination of coronal sections. Locations of cannula tips were superimposed on reference brains. Numbers (given in millimeters) represent the distance from the bregma, according to The Mouse Brain in Stereotaxic Coordinates by Franklin and Paxinos.

(**G**) Temporal changes of cutaneous temperature during 20 min of 2MT presentation (left) and the mean cutaneous temperature changes are indicated (right). The cutaneous temperature change was calculated as the change from the onset of 2MT presentation (n = 6 each).

Data are means ± SEM. Student's t-test was performed between saline and each condition. $^{**}p<0.01$; ns, $p>0.05$.

Supplementary Figure 9. Full uncropped gel image for Fig. 4f

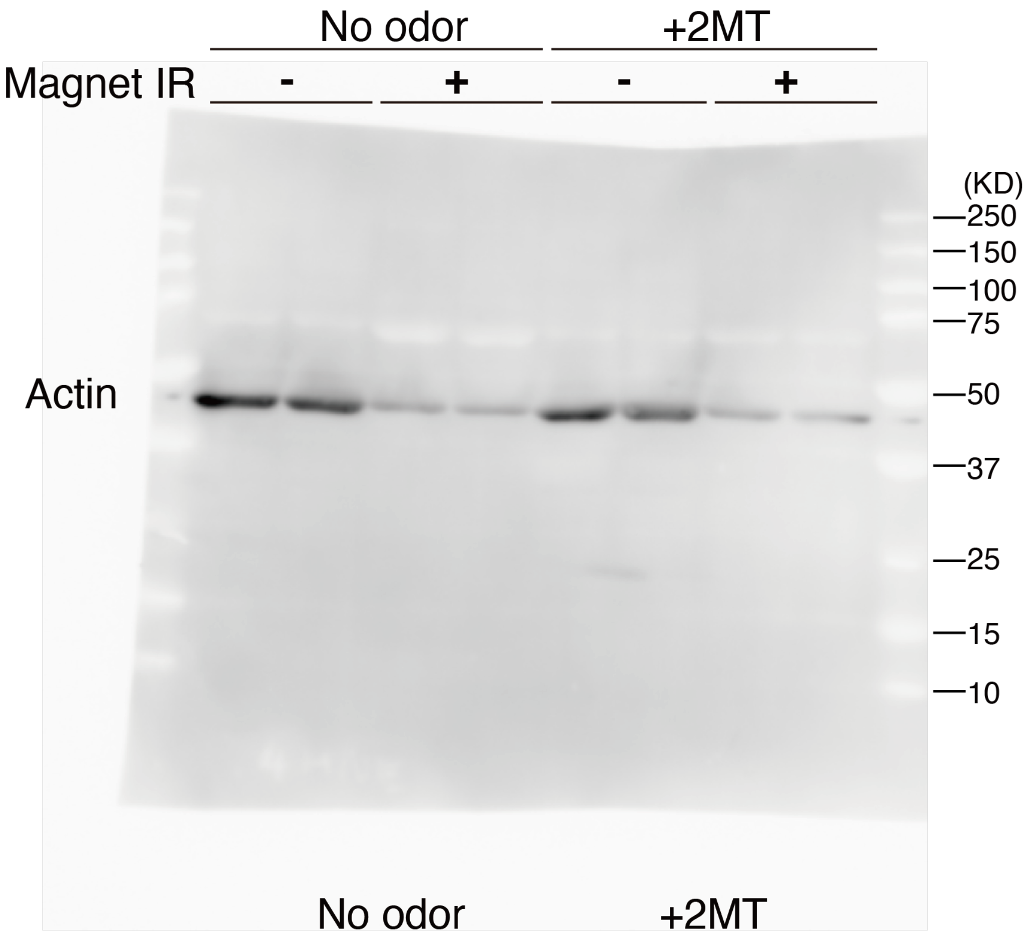

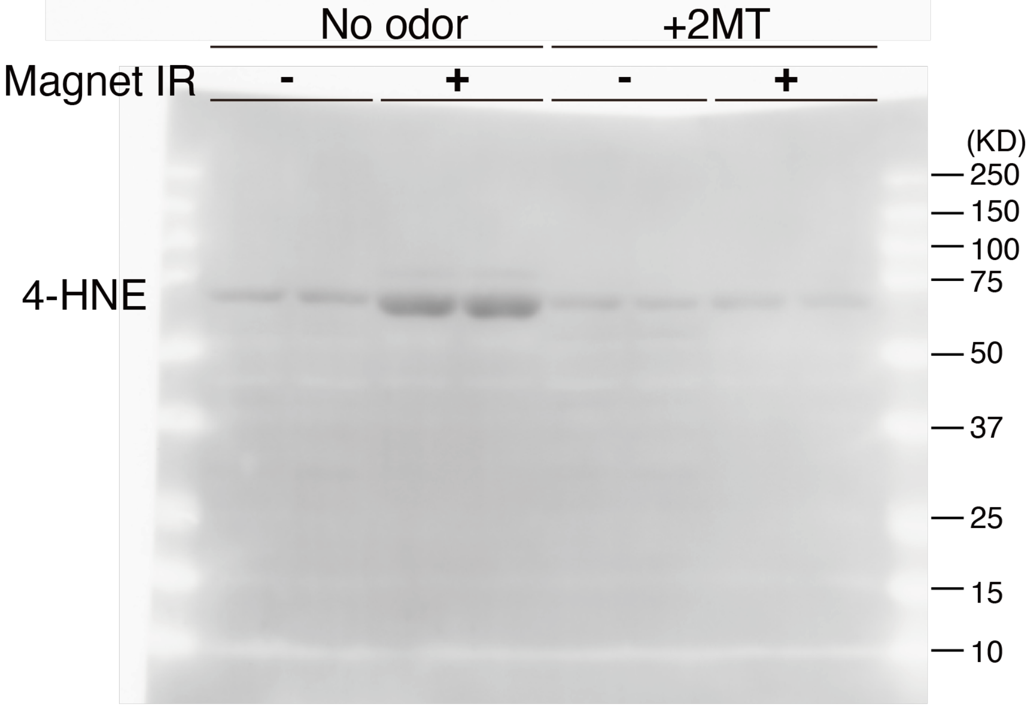

Supplementary References

1 Pulga, A., Porte, Y. & Morel, J. L. Changes in C57BL6 Mouse Hippocampal Transcriptome Induced by Hypergravity Mimic Acute Corticosterone-Induced Stress. *Front Mol Neurosci* **9**, 153, doi:10.3389/fnmol.2016.00153 (2016).

2 Graf, E. N. *et al.* Corticosterone acts in the nucleus accumbens to enhance dopamine signaling and potentiate reinstatement of cocaine seeking. *J Neurosci* **33**, 11800-11810, doi:10.1523/JNEUROSCI.1969-13.2013 (2013).

3 Isosaka, T. *et al.* Htr2a-Expressing Cells in the Central Amygdala Control the Hierarchy between Innate and Learned Fear. *Cell* **163**, 1153-1164, doi:10.1016/j.cell.2015.10.047 (2015).
