# Supplementary material for: Artificial hibernation/life-protective state induced by thiazoline-related innate fear odors
Source: Commun Biol. 2021 Jan 22;4:101. doi: 10.1038/s42003-020-01629-2 (PMC7822961; doi:10.1038/s42003-020-01629-2)
Supplement: Supplementary file 3 — Description of Supplementary Files [file 42003_2020_1629_MOESM3_ESM.pdf]

Description of Additional Supplementary Files

File name: Supplementary Video1
Description: Cutaneous temperature changes in response to innate versus learned fear odors. Cutaneous temperature changes in response to 2MT and a conditioned odor (anisole) paired with foot shock was monitored by infrared camera.

File name: Supplementary Data 1
Description: Source data file. Source data underlying plots shown in figures.
