# Supplementary material for: Artificial hibernation/life-protective state induced by thiazoline-related innate fear odors
Source: Commun Biol. 2021 Jan 22;4:101. doi: 10.1038/s42003-020-01629-2 (PMC7822961; doi:10.1038/s42003-020-01629-2)
Supplement: Supplementary file 6 — Reporting Summary [file 42003_2020_1629_MOESM6_ESM.pdf]

Corresponding author(s): Ko Kobayakawa, Ph.D.

Last updated by author(s): Dec 8, 2020

Reporting Summary

Nature Research wishes to improve the reproducibility of the work that we publish. This form provides structure for consistency and transparency in reporting. For further information on Nature Research policies, see our Editorial Policies and the Editorial Policy Checklist.

Statistics

For all statistical analyses, confirm that the following items are present in the figure legend, table legend, main text, or Methods section.

n/a	Confirmed	
☐	☒	The exact sample size (*n*) for each experimental group/condition, given as a discrete number and unit of measurement
☐	☒	A statement on whether measurements were taken from distinct samples or whether the same sample was measured repeatedly
☐	☒	The statistical test(s) used AND whether they are one- or two-sided<br>*Only common tests should be described solely by name; describe more complex techniques in the Methods section.*
☒	☐	A description of all covariates tested
☐	☒	A description of any assumptions or corrections, such as tests of normality and adjustment for multiple comparisons
☐	☒	A full description of the statistical parameters including central tendency (e.g. means) or other basic estimates (e.g. regression coefficient) AND variation (e.g. standard deviation) or associated estimates of uncertainty (e.g. confidence intervals)
☐	☒	For null hypothesis testing, the test statistic (e.g. *F*, *t*, *r*) with confidence intervals, effect sizes, degrees of freedom and *P* value noted<br>*Give P values as exact values whenever suitable.*
☒	☐	For Bayesian analysis, information on the choice of priors and Markov chain Monte Carlo settings
☒	☐	For hierarchical and complex designs, identification of the appropriate level for tests and full reporting of outcomes
☒	☐	Estimates of effect sizes (e.g. Cohen's *d*, Pearson's *r*), indicating how they were calculated

Our web collection on statistics for biologists contains articles on many of the points above.

Software and code

Policy information about availability of computer code

Data collection	TH92-707 data capture program 1.2 (J) (NEC Avio Infrared Technologies) for collection of cutaneous body temperature; Dataquest A.R.T. 4.30 (DSI) for collection of core body temperature and heart rate; ARCO2000 NET 3.51 (ARCO System) for collection of oxygen consumption; NDP.scan 2.5 and NDP.view2 (Hamamatsu Photonics) for imaging c-fos stained sections; LAS AF 2.7.0.9329 (Leica) and BZ-II Viewer (Keyence) for fluorescent images; Image Reader Las-4000 (Fujifilm) for detection of Western blot signals.
Data analysis	C-fos expression level was counted by single blinded investigator. Video-recorded locomotor activities were converted to AVI file using Virtual lab and analyzed using ImageJ 1.50i and trajr R package. Statistical analysis was performed with GraphPad Prism 8 and Microsoft Excel.

For manuscripts utilizing custom algorithms or software that are central to the research but not yet described in published literature, software must be made available to editors and reviewers. We strongly encourage code deposition in a community repository (e.g. GitHub). See the Nature Research guidelines for submitting code & software for further information.

Data

Policy information about availability of data

All manuscripts must include a data availability statement. This statement should provide the following information, where applicable:

- Accession codes, unique identifiers, or web links for publicly available datasets
- A list of figures that have associated raw data
- A description of any restrictions on data availability

All data generated or analysed during this study are included in this published article (and its supplementary information files).

Field-specific reporting

Please select the one below that is the best fit for your research. If you are not sure, read the appropriate sections before making your selection.

☒ Life sciences ☐ Behavioural & social sciences ☐ Ecological, evolutionary & environmental sciences

For a reference copy of the document with all sections, see nature.com/documents/nr-reporting-summary-flat.pdf

Life sciences study design

All studies must disclose on these points even when the disclosure is negative.

Sample size	Sample size was chosen on the basis of pilot experiments and according to previously reported publications done in the field.
Data exclusions	No data were excluded from the analyses.
Replication	Experiments were performed with sufficient animals to demonstrate statistical significance. All attempts at replication were successful.
Randomization	We used the mice for the experiments at random.
Blinding	The c-fos expression analysis was performed by single-blinded experimenter.

Reporting for specific materials, systems and methods

We require information from authors about some types of materials, experimental systems and methods used in many studies. Here, indicate whether each material, system or method listed is relevant to your study. If you are not sure if a list item applies to your research, read the appropriate section before selecting a response.

Materials & experimental systems

n/a	Involved in the study	
☐	☒	Antibodies
☒	☐	Eukaryotic cell lines
☒	☐	Palaeontology and archaeology
☐	☒	Animals and other organisms
☒	☐	Human research participants
☒	☐	Clinical data
☒	☐	Dual use research of concern

Methods

n/a	Involved in the study	
☒	☐	ChIP-seq
☒	☐	Flow cytometry
☒	☐	MRI-based neuroimaging

Antibodies

Antibodies used	anti-cleaved caspase-3 antibody; 1:500; Cell Signaling Technology<br><br>anti-MAP-2 antibody; 1:500; Merck Millipore<br><br>anti 4-HNE Ab antibody; 1:5000, Abcam<br><br>anti-actin Ab; 1:5000; Abcam<br><br>anti-DIG antibody; 1:500; Jackson ImmunoResearch
Validation	All antibodies are commercial and have been validated.

Animals and other organisms

Policy information about studies involving animals; ARRIVE guidelines recommended for reporting animal research

Laboratory animals	Male adult C57BL/6 mice; Male adult Ucp1 KO (JAX#17476) mice.<br>All mice were 9-13 weeks old at the start of experiment.
Wild animals	The study did not involve wild animals.

Field-collected samples	The study did not involve samples collected from the field.
Ethics oversight	The animal experimental procedures were in accordance with the Kansai Medical University and use of laboratory animals were approved by the Animal Research Committee of Kansai Medical University.

Note that full information on the approval of the study protocol must also be provided in the manuscript.
